# Trachoma

**Published:** 1919

**Authors:** 


***Trachoma.*** By E. H. Cary. *Journal of the American Medical Association,* Nov. 16, 1918.

The author says that he has operated on more than 500 cases of trachoma, and has reached a point at which he can say to the patient that he will cure him in from three to six weeks, and in few cases has there been a relapse. Sometimes other conditions exist, preventing this rapid cure, such as diseases of the turbinals in the nose, removal of which will cure the eye condition. Occasionally this is confined to one eye, but he has seen both affected. In the few cases in which the repair operation has failed, there is a distinct reason traceable to a definite cause, removal of which abates the symptoms. The histologic condition of the lid is described, as the operative procedure cannot succeed without a clear understanding of the pathologic conditions developing.

There are three distinct features characterizing trachoma : granulation, papillary hypertrophy, and the formation of connective tissue. Callan's classification is adopted by the author, including its fourth stage of cicatricial tissue. As is generally known, the expression operation is of value and cures many cases seen early when pathologic conditions are still limited to the conjunctiva.

But when pannus appears along with any other corneal disturbance, Cary says, we may know that we have pathologic changes in the tarsal region and other structures, mechanically irritating the cornea.

The cause of the crimping seen while operating is explained by the tendency of the levator to roll on itself and to lessen the amount of tarsus in evidence after the removal. The action of the involuntary orbicularis muscle is to keep the lid closed in sleep, and possibly to lift some of the weight of the lid of the globe. The inflammation of the connective tissue between the tarsus and the palpebral portion of the orbicularis increases the fibrous connective tissue, and this fibrosis extends in between its fibers, shortening it and accentuating the already present crimping of the tarsus, keeping up the irritation. By observing the lid, we can see how the pathologic condition varies in different cases, causing blocking of the meibomian glands in some, calcareous deposits in others, etc. Splitting the margin of the lid is necessary in some cases to overcome the crimping, and particularly the torsion of the cilia. Tarsal resection of any degree is a kind of repair of the pathologic changes : and less tarsal surface is offered for muscular attachment, compensating thus for loss of muscular tissue.

In a broad sense, Cary says, his repair operation is an adaptation of methods previously used for entropion, with the distinct difference that there is a pathologic condition to correct, calling for the removal of muscular and fibrous tissue, the breaking up of fibrous bands, and the pushing back of the levator tendon with a probable flattening of the tarsus. Whether the splitting of the margin of the lid is to be done is determined during the operation, according to the operator's judgment of his control of the position of the cilia and the skin surface to be stitched. "The upper portion of the skin may be removed or not, as he feels the need of pinning the skin surface to the upper portion of the tarsus. This constitutes the fundamental procedure in tarsal repair. Of course, the expression of all granules is the first thing to be done before the lid operation is begun, as heretofore described."

